# Dual role of α7 nicotinic acetylcholine receptors in the retrosplenial cortex for aversive memory acquisition and retrieval

**DOI:** 10.3389/fnbeh.2024.1359729

**Published:** 2024-01-26

**Authors:** Verónica Pastor, Cynthia Katche

**Affiliations:** ^1^CONICET-Universidad de Buenos Aires, Instituto de Biología Celular y Neurociencia “Prof. Eduardo De Robertis”, Buenos Aires, Argentina; ^2^Departamento de Ciencias Fisiológicas, Facultad de Medicina, Universidad de Buenos Aires, Buenos Aires, Argentina; ^3^Instituto Tecnológico de Buenos Aires, Buenos Aires, Argentina

**Keywords:** posterior cingulate cortex, inhibitory avoidance, methyllycaconitine, long-term memory, cholinergic system, Alzheimer’s disease, encoding, retrieval

## Abstract

In the retrosplenial cortex (RSC), the role of cholinergic modulation via α7 nicotinic receptors and their involvement in memory is unknown. In recent years, the RSC has been shown to deteriorate in the early stages of Alzheimer’s disease (AD). Likewise, the cholinergic system has been postulated as one of those responsible for cognitive impairment in patients with AD. Great interest has arisen in the study of α7 nicotinic receptors as more specific targets for the treatment of this disease. For this reason, we aim to study the role of α7 receptors of the RSC in memory processing. We infused a selective α7 receptor antagonist into the anterior part of the RSC (aRSC) to assess its role in different phases of aversive memory processing using an inhibitory avoidance task. We found that α7 nicotinic receptors are involved in memory acquisition and expression, but not in its consolidation. These results identify aRSC α7 nicotinic receptors as key players in aversive memory processing and highlight their significant potential as therapeutic targets for Alzheimer’s disease.

## Introduction

The retrosplenial cortex (RSC) is essential for learning and memory processing (Corcoran et al., 2018; Stacho and Manahan-Vaughan, 2022). In rodents, the retrosplenial cortex comprises the entire posterior cingulum. In humans, it comprises only Broadman’s areas 29 and 30 (Vogt and Peters, 1981). During the last decades, several lesion and neuroimaging studies demonstrated its requirement and activation in memory tasks in humans (Valenstein et al., 1987; Maguire, 2001) and rodents (Sutherland et al., 1988; Vann and Aggleton, 2002, 2004). In particular, our studies in the inhibitory avoidance task highlighted the relevance of the anterior part of the RSC (aRSC) for the consolidation and retrieval of aversive memories (Katche et al., 2013).

Mostly, the RSC has attracted attention in recent years because it is affected in the early stages of Alzheimer’s disease (AD) (Pengas et al., 2010; Aggleton, 2014; Tu et al., 2015) and cognitive impairments associated with aging (Trask and Fournier, 2022). The cholinergic system has been postulated as one of those responsible for cognitive impairment in patients with AD (Hampel et al., 2018; Hoskin et al., 2019; Koukouli and Changeux, 2020). In this sense, the study of pharmacological strategies aimed at modifying cholinergic tone such as acetylcholinesterase inhibitors has dominated preclinical and clinical studies in the field of AD. In fact, in a rodent model of AD, deposits of the ABeta peptide have been described in cholinergic axons innervating the RSC (Robertson et al., 2009).

The RSC is known to receive cholinergic inputs from the basal forebrain (Bigl et al., 1982; Mesulam et al., 1983). However, the role of RSC cholinergic modulation in memory processing is yet to be elucidated. Among ionotropic nicotinic acetylcholine receptors (nAChRs), the α7 subtype is one of the most prominent in the central nervous system. Compared with other nAChR subtypes, α7 nAChR has the highest calcium permeability, which make these receptors key players in plasticity processes, such as long-term potentiation (LTP), and then, in learning and memory (Koukouli and Changeux, 2020; Pastor and Medina, 2023). Indeed, several reports pointed out α7 nAChRs as promising pharmacological targets for cognitive improvement in neurological disorders such as AD (see for references Greenfield et al., 2022). α7 nAChRs are expressed in the RSC, mainly in gabaergic interneurons (Murakami et al., 2013). However, whether their activation is required for memory processing is largely unknown. Here, we studied in the aRSC the effect of the infusion of methyllycaconitine (MLA), a selective antagonist of α7 nAChRs, on different phases of an aversive memory in adult rats. Our results show that α7 nAChRs in the aRSC are differentially involved in the acquisition, consolidation, or retrieval of an aversive memory.

## Materials and methods

### Subjects

Experiments were conducted in male Wistar rats (UBA, Argentina) weighting 220–250 g. Animals were housed three to a cage and kept at a constant temperature of 23°C, with water and food *ad libitum*, under a 12-h light/dark cycle (lights on at 7:00 am). Experimental procedures were performed in accordance with the USA National Institutes of Health Guide for the Care and Use of Laboratory Animals and were approved by the Animal Care and Use Committees of the University of Buenos Aires (CICUAL).

### Surgery

Animals were anesthetized with a mix of ketamine (80 mg/kg) and xylazine (10 mg/kg) and placed on a stereotaxic frame. The skull was exposed and leveled (flat skull, lambda and bregma at the same elevation). 22-G guide cannulas were bilaterally implanted, aimed to the aRSC: AP −3.9 mm/LL ± 0.5 mm/DV −1.8 mm (from Bregma; Paxinos and Watson, 2007). Cannulas were fixed to the skull with dental acrylic and protected with modified 30-G needles. Immediately after surgery, animals were injected with meloxicam (0.2 mg/kg) as analgesic and gentamicin (2 mg/kg) as antibiotic. Animals were allowed to recover from surgery for 5–7 days before any experimental manipulation.

### Drug administration

Infusions were delivered through an injector cannula extending 1 mm beyond the tip of the guide cannula connected to 10 μL Hamilton syringes. The volume infused was 1 μl per side and the infusion rate was 1 μl/min. The injector was left in place for an additional minute after infusion to allow diffusion and to prevent reflux. Dose was as follows: Methyllycaconitine Citrate (MLA; Abcam#ab120072) was dissolved in sterile saline solution and administered bilaterally intra-aRSC (5 μg/μl/hemisphere). The same volume of vehicle (1 μl/hemisphere) was used in control rats. Indicated dose was based on the molecular weight of the salt (MLA) and was determined based on preliminary studies and previous reports (Chan et al., 2007; Pastor et al., 2021, 2023). After completing the behavioral procedures, the positioning of the cannula was confirmed by the aRSC infusion of 1 μl of 4% methylene blue in saline. The extension of the dye was taken as indicative of the diffusion of the drug, as previously published (de Landeta et al., 2022). Only animals with both cannulae in the correct place were included in the analysis.

### Inhibitory avoidance task

After recovery from surgery, animals were handled once a day for two days and then trained in the inhibitory avoidance task (IA) as described previously (Bekinschtein et al., 2007). Briefly the apparatus was a 50 cm × 25 cm × 25 cm acrylic box whose floor was a grid made of 1 mm-caliber steel bars. The left end of the grid was covered with a 7 cm-wide, 5 cm-high acrylic platform. Rats were infused into the aRSC with MLA or vehicle 15 min before the training session, immediately after the training session, or 40 min before the test, depending on the experiment. For training, animals were placed on the platform and as they stepped down onto the grid received a single 3-sec, 0.5 mA scrambled footshock. Rats were tested for retention 24 h after training. All animals were tested only once. In the test sessions the footshock was omitted. The time spent on the platform during this session was taken as an indicator of retention.

### Open field (OF)

Locomotor activity was assessed during 5 min in an open field arena (50 cm × 50 cm × 39 cm) which had black walls and floor divided into nine squares by white lines. Rats were infused into the aRSC with MLA or vehicle 15 min before being placed in the open field. The number of line crossings and the number of entries to the central square were measured.

### Elevated plus maze (EPM)

Immediately after the OF test, anxiety-like behavior was assessed in the same group of rats using a maze elevated 65 cm over the floor, which consisted of two open (45 cm length × 10 cm width) and two closed arms (45 cm length × 10 cm width × 50 cm height) opposite to each other and inter-connected by a central platform (10 cm × 10 cm). Each animal was placed in the center, facing an open arm, and left in the maze for 5 min. The percentage of time spent in open arms and the number of entries to open arms were used as indices of anxiety, whereas the total number of entries to open and closed arms were used as an index of general locomotion.

### Simultaneous oddity discrimination task (SOD)

The SOD task was conducted in an independent group of rats to evaluate if the infusion of the α7 nAChR antagonist affected attention and perception (Bekinschtein et al., 2013). The exploration area was triangular in shape with high, homogenous white walls constructed from Perspex to prevent the rat from looking out into the room. All walls were 60 cm wide and 60 cm tall, and the objects were placed 2.50 cm apart along the back wall during the test session. All rats were habituated to the empty apparatus for 10 min in which they were allowed to freely explore it. For the habituation session, the rat was placed in the triangular apparatus, facing the corner of the triangle away from the back wall. Test session began 24 h after the habituation session. MLA and vehicle infusions were made 15 min before the test. All object sets (2 repeated and 1 different) were placed in the back wall of the apparatus before the rat was placed. The rat was placed in the corner of the triangular apparatus (away from the objects). The time spent exploring the three objects during a testing phase was scored for 5 min by an experimenter viewing the rat. Exploration of an object was defined as directing the nose to the object at a distance of < 2 cm and/or touching it with the nose. A SOD index was calculated as follows: [time of exploration of the different object–(time of exploration of similar objects/2)]/total time of object exploration.

### Hot plate test

The hot plate test was used in an independent group of rats to determine the potential analgesic activity induced by the antagonism of α7 nAChRs in the aRSC. MLA was infused 15 min before placing the rats on the hot plate until a nociceptive response (i.e., paw withdrawal) was observed. An observer blind to the treatment measured the latency of paw withdrawal using a manual stopwatch. The temperature of the hot plate was set and calibrated at 48°C.

### Data analysis

Data was analyzed by Student’s *t*-test, Mann–Whitney or Wilcoxon signed rank test as appropriate. For simplicity, all the results are presented as mean ± SEM and each point represents an individual animal. GraphPad Outliers Grubbs’ test with alpha 0.05 was used to assess the presence of outliers. All experiments were repeated twice to assess reproducibility which was achieved in all cases. Significant differences were set at *p* < 0.05.

## Results

### α7 nAChRs involvement in IA memory encoding

**FIGURE 1:**
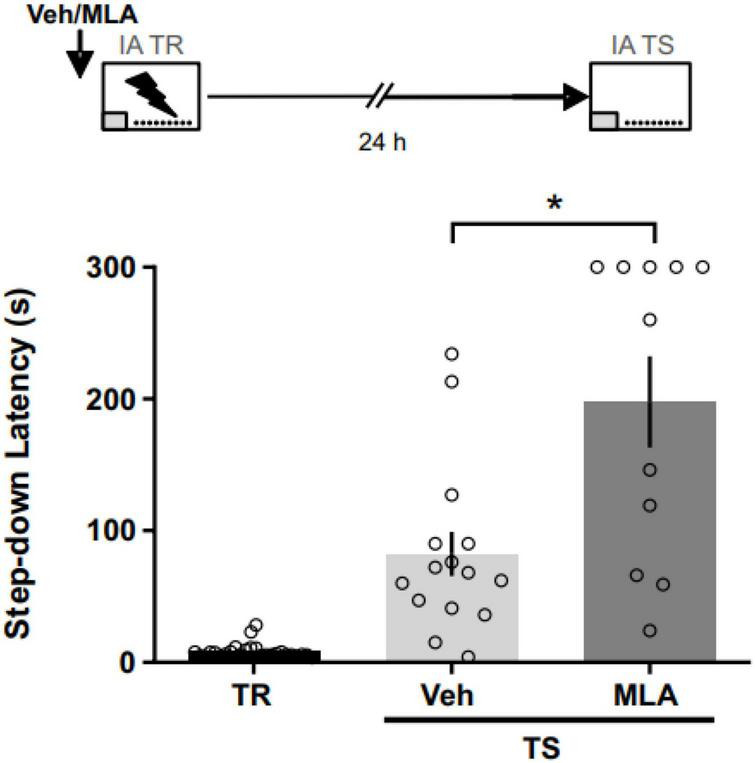
α7 nAChRs antagonism in the aRSC before IA training improved long-term memory expression. Data are expressed as the mean ± SEM of the latency to step down during training (TR) or 24 h test (TS) for rats infused with methyllycaconitine (MLA) or vehicle (Veh) 15 min before TR. Mann–Whitney test; *p < 0.05; *n* = 11–15.

To assess the role of aRSC α7 nAChRs during the acquisition phase of an aversive memory, we infused MLA in the aRSC 15 min before the IA training (Figure 1). We found longer latency to step down compared to controls at 24 h in MLA-infused rats (Mann–Whitney test; *U* = 36; *p* = 0.014; n_*VEH*_ = 15; n_*MLA*_ = 11), suggesting that aRSC α7 nAChRs are involved in IA memory encoding. Three rats were excluded from the analysis due to cannulae misplacement.

### α7 nAChRs are not involved in IA memory consolidation

**FIGURE 2:**
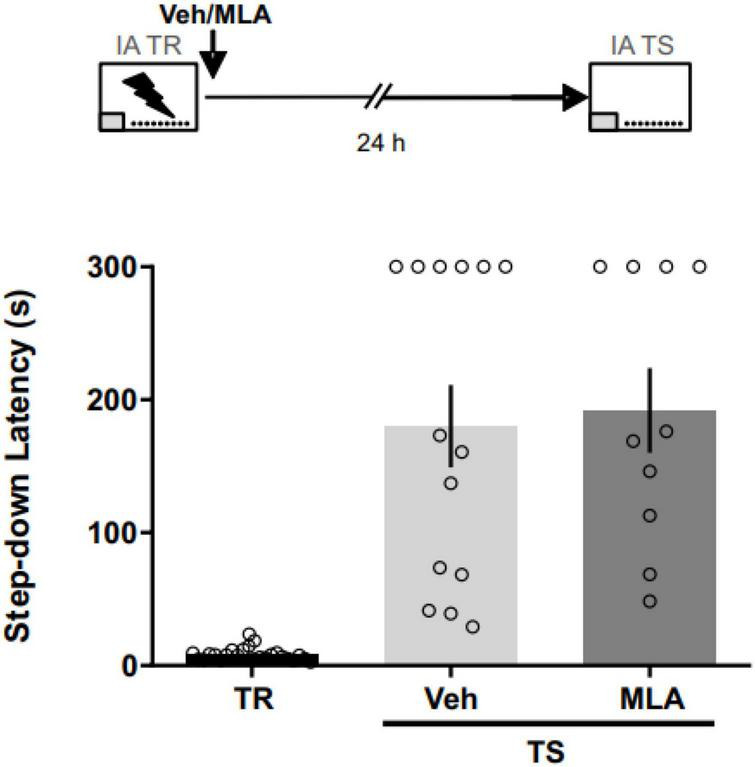
α7 nAChRs antagonism in the aRSC did not affect IA memory consolidation. Data are expressed as the mean ± SEM of the latency to step down during training (TR) or 24 h test (TS). No significant differences were found between rats infused with methyllycaconitine (MLA) or vehicle (Veh) immediately after TR; Mann–Whitney test, *n* = 10–14.

To assess the role of aRSC α7 nAChRs in the consolidation phase of an aversive memory, we infused MLA in the aRSC immediately after the IA training (Figure 2). We found no differences between MLA-infused rats and vehicle controls at 24 h test (Mann–Whitney test; *U* = 63; *p* = 0.687; n_*VEH*_ = 14; n_*MLA*_ = 10), suggesting no involvement of aRSC α7 nAChRs in IA memory consolidation. Two rats were excluded from the analysis due to cannulae misplacement.

### α7 nAChRs are necessary for IA memory expression

**FIGURE 3:**
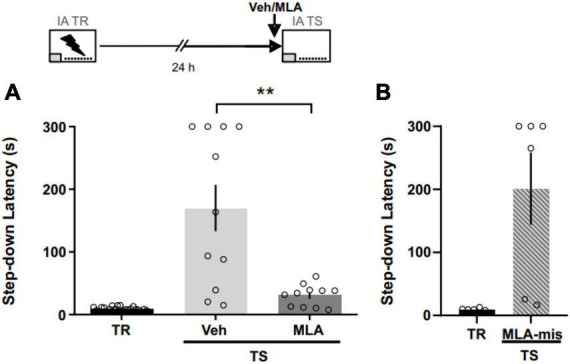
α7 nAChRs antagonism in the aRSC blocked IA memory expression. **(A)** Data are expressed as the mean ± SEM of the latency to step down during training (TR) or 24 h test (TS) for rats infused with methyllycaconitine (MLA) or vehicle (Veh) 40 min before TS. Mann Whitney test; **p < 0.01; *n* = 11. **(B)** Data are expressed as the mean ± SEM of the latency to step down during training (TR) or 24 h test (TS) for rats infused with methyllycaconitine (MLA) with cannulae misplacement.

To assess the role of aRSC α7 nAChRs in IA memory expression, we infused MLA before the 24 h test (Figure 3). We found that MLA shortened latency to step down compared to controls (Figure 3A; Mann–Whitney test; *U* = 17; *p* = 0.0030; *n* = 11 per group), supporting the role of aRSC α7 nAChRs in aversive memory retrieval. Six rats were excluded from the analysis due to cannulae misplacement. In support of our results, in those animals with cannulae misplacement (Figure 3B), MLA did not show any memory blocking effect, evidenced as an increase in the latency to step-down in the test compared to the training (Wilcoxon one-tailed signed rank test; *W* = 21; *p* = 0.015; *n* = 6).

### The antagonism of α7 nAChRs does not affect locomotor activity and anxiety-like behavior

**FIGURE 4:**
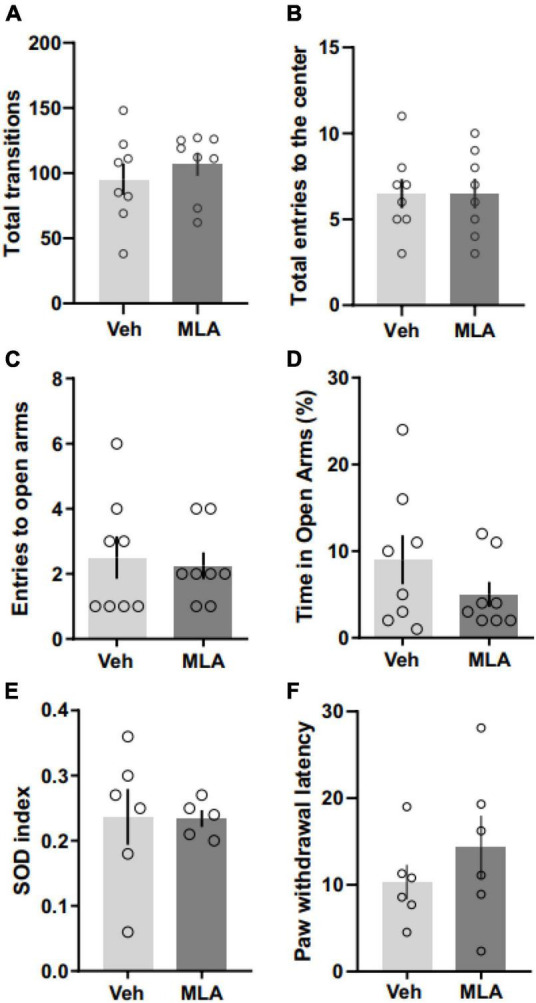
α7 nAChRs antagonism in the aRSC did not affect locomotor activity, anxiety-like behavior, attention, perception or nociception. Data are expressed as the mean + SEM of the total number of transitions **(A)** or the number of entries to the center zone **(B)** of an open field; the number of entries to the open arms **(C)** and the percentage of time spent in open arms **(D)** of the elevated plus maze; the SOD index **(E)** or the latency to paw withdrawal in the hot plate test **(F)**. No significant differences were found by Student’s *t*-test between rats infused with methyllycaconitine (MLA) or vehicle (Veh) 15 min before the behavioral test (*n* = 5–8).

To investigate if the antagonism of aRSC α7 nAChRs may induce any deficit on locomotor activity which could interfere with our results, we performed an OF test following the infusion of MLA in the aRSC. We found no differences between MLA-infused rats and vehicle controls in the total number of crossings [Figure 4A; Student’s *t*-test; *t*(14) = 0.77; *p* = 0.456] nor in the number of entries to the center zone [Figure 4B; Student’s *t*-test; *t*(14) = 0; *p* > 0.999; *n* = 8 per group]. To assess the effect of the antagonism of α7 nAChRs on the anxiety-like state in rats infused in the aRSC, we performed an EPM test following MLA administration. We found no differences in the total number of entries to the open arms [Figure 4C; Student’s *t*-test; *t*(14) = 0.323; *p* = 0.751; *n* = 8 per group] nor in the percentage of time in open arms [Figure 4D; Student’s *t*-test; *t*(14) = 1.263; *p* = 0.227; *n* = 8 per group] between MLA-infused rats and vehicle controls. Total entries to open + closed arms were quantified to assess locomotor activity during the EPM test and showed no differences between groups [11.5 ± 2.8 for vehicle; 12.4 ± 1.5 for MLA; Student’s *t*-test: *t*(14) = 0.276; *p* = 0.786; *n* = 8 per group].

### The antagonism of α7 nAChRs did not affect attention and sensory perception

Considering that our results could be influenced by differences in attention or sensory perception, we decided to assess if the infusion of MLA in the aRSC had any effect on the simultaneous oddity discrimination task or on the hotplate test. We found no differences between MLA and vehicle infused rats in the SOD task (Figure 4E; Mann–Whitney test; *U* = 12; *p* = 0.628; *n* = 5–6) nor in the hotplate test [Figure 4F; Student’s *t*-test; *t*(10) = 0.9633; *p* = 0.358; *n* = 6], suggesting that the antagonism of α7 nAChRs in the aRSC did not affect sensory perception nor attention.

## Discussion

In this study, we demonstrated for the first time that aRSC α7 nAChRs are critically involved in the processing of information related to fearful experiences. By using a one-trial IA task and local infusion of MLA in the aRSC, we found that α7 nAChRs are differentially involved in the acquisition, consolidation, and expression phases of an aversive memory.

The aRSC is necessary for aversive memory processing (Katche et al., 2013). However, the cellular and molecular mechanisms underlying its function are not fully understood. Different neurotransmitter systems are likely involved in memory processing in the aRSC. Given the importance of the RSC and of the cholinergic system, specifically the α7 nAChR subtype, on AD pathogenesis, we focused on the study of those receptors in memory processing.

We first asked if α7 nAChR activation was necessary for the acquisition of an aversive memory in the aRSC. Our results showed that the antagonism of α7 nAChRs in the aRSC during memory acquisition led to an improvement in memory expression the day after, supporting their involvement in memory encoding. Due to the lack of information about the role of α7 nAChRs in the aRSC, this finding could be interpreted in different ways. Considering that attention is required for memory encoding (Muzzio et al., 2009), aRSC α7 nAChRs role in IA memory encoding may be related to their involvement in attention and not only in memory processing (Ballinger et al., 2016). Thus, we performed a control experiment to assess the effect of infusing MLA in the aRSC before the SOD test. Our results suggest that aRSC α7 nAChRs are not involved in attention processing. Another possibility is that aRSC α7 nAChRs may be modulating how sensory information is perceived when the shock is received during the training session. To assess this possibility, we performed a hot plate test following the infusion of MLA. We did not find any effect suggesting that aRSC α7 nAChRs are not involved in sensory perception.

It is noteworthy that when MLA is infused before training, it likely remains in the brain during and after the training session (Turek et al., 1995). This makes it challenging to determine if MLA effects on memory result from enhancing encoding or affecting early consolidation processes. Therefore, we performed another experiment where we administered MLA immediately after the training session. We found no changes in memory expression, supporting the idea that α7 nAChRs are involved in the encoding but not the consolidation phase of IA memory. This is consistent with previous research showing a differential role of α7 nAChRs in encoding but not consolidation of rewarding memory in the medial prefrontal cortex (Pastor et al., 2021), highlighting that cortical α7 nAChRs have an essential role in the acquisition of different types of memories.

Previous reports have shown that the RSC is involved in the retrieval of aversive memories (Fournier et al., 2021), including those assessed by the IA task (Katche et al., 2013). Thus, we aimed to investigate the potential involvement of the cholinergic system, specifically through α7 nAChRs, in mediating that function. Differently from what we reported for the medial prefrontal cortex, where α7 nAChRs are not involved in aversive memory expression (Pastor et al., 2023), here we found that α7 nAChRs activation in the aRSC is critical for IA memory expression. These findings identify the aRSC as a key brain area where cholinergic modulation through α7 nAChRs influences aversive memory expression.

There is still debate about how the RSC participates in aversive memory processing. Prior studies using fear conditioning protocols showed that the RSC is involved in the retrieval but not in the acquisition of the aversive memory (Fournier et al., 2021). However, an increased activation of the RSC following mild tail shock paired with tactile stimuli was evidenced in mice by assessing the levels of c-fos expression (Radwanska et al., 2010), supporting its role in the acquisition of aversive classical associative learning. Thus, it is likely that different cellular and molecular mechanisms are involved in different phases of aversive memory processing in the RSC. Regarding α7 nAChRs, there is scarce information in the literature about their localization and function in the RSC. It has been described that α7 nAChRs are mainly expressed in RSC GABAergic neurons (Murakami et al., 2013). Thus, the activation of these receptors is likely to modulate local neuronal activity in a complex manner. At a synaptic level, inactivation of α7 nAChRs may facilitate LTP induction as was reported in the hippocampus (Fujii et al., 2000; Miguelez Fernández et al., 2021), thus facilitating memory acquisition. Further research is needed to assess this possibility.

In conclusion, our results suggest a dual effect of α7 nAChRs in the aRSC, highlighting their dynamic involvement in different stages of memory processing. It remains elusive the mechanisms by which α7 nAChRs modulation differentially affects encoding and retrieval of the IA memory and further research is needed to uncover the processes that underlie the observed dual effect. Elucidating these mechanisms will not only enhance our understanding of α7 nAChRs modulation in the aRSC, but also enhance the development of targeted interventions for memory-related disorders.

## Data availability statement

The raw data supporting the conclusions of this article will be made available by the authors, without undue reservation.

## Ethics statement

The animal study was approved by the Animal Care and Use Committee of the University Buenos Aires (CICUAL). School of Medicine, University of Buenos Aires. The study was conducted in accordance with the local legislation and institutional requirements.

## Author contributions

VP: Conceptualization, Funding acquisition, Investigation, Writing – original draft. CK: Conceptualization, Funding acquisition, Investigation, Writing – original draft.
